# Retraction: Fecal Microbiota Transplantation (FMT) in Clostridium difficile Infection: A Paradigm Shift in Gastrointestinal Microbiome Modulation

**DOI:** 10.7759/cureus.r206

**Published:** 2025-11-20

**Authors:** Muhammad Hamza Saeed, Sundas Qamar, Ayesha Ishtiaq, Qudsia Umaira khan, Asma Atta, Maryam Atta, Hifza Ishtiaq, Marriam Khan, Muhammad Rawal Saeed, Ayesha Iqbal

**Affiliations:** 1 Internal Medicine, Russells Hall Hospital, Dudley, GBR; 2 Geriatrics, Russells Hall Hospital, Dudley, GBR; 3 Internal Medicine, Foundation University Medical College, Islamabad, PAK; 4 Physiology, Combined Military Hospitals (CMH) Lahore Medical College and Institute of Dentistry (IOD), Lahore, PAK; 5 Surgery, Azad Jammu and Kashmir (AJK) Medical College, Muzaffarabad, PAK; 6 Medicine, Abbas Institute of Medical Sciences, Muzaffarabad, PAK; 7 Research, Hamdard Hospital, Karachi, PAK; 8 Trauma and Orthopedics, Rawalpindi Medical University/Benazir Bhutto Hospital, Rawalpindi, PAK; 9 Medicine, Sharif Medical and Dental College, Lahore, PAK

The Editors-in-Chief have retracted this article following the identification of a major inconsistency discovered during post-publication review. Although the study focuses on fecal microbiota transplantation for C. difficile infection, the discussion unexpectedly shifts to an unrelated comparison of anti-diabetic medications, rendering the article scientifically unreliable. As this inconsistency undermines the credibility and coherence of the work, the article has been retracted. The authors disagree with the decision to retract.

